# Genome Sequence of a Single-Stranded DNA Virus Identified in Gila Monster Feces

**DOI:** 10.1128/MRA.00925-18

**Published:** 2018-08-23

**Authors:** Vasishta Somayaji, Dale DeNardo, Melissa A. Wilson Sayres, Mellecha Blake, Kara Waits, Rafaela S. Fontenele, Simona Kraberger, Arvind Varsani

**Affiliations:** a Biodesign Center for Fundamental and Applied Microbiomics, Arizona State University, Tempe, Arizona, USA; b School of Life Sciences, Arizona State University, Tempe, Arizona, USA; c Center for Evolution and Medicine, Arizona State University, Tempe, Arizona, USA; d Structural Biology Research Unit, Department of Clinical Laboratory Sciences, University of Cape Town, Observatory, Cape Town, South Africa; Queens College

## Abstract

The Gila monster (Heloderma suspectum) is native to the Sonoran Desert. Metagenomic analyses of a Gila monster fecal sample revealed the presence of a small, circular, single-stranded DNA virus that is most closely related to a gemykrogvirus (family Genomoviridae) genome from caribou feces sharing 88% genome-wide pairwise identity.

## ANNOUNCEMENT

The Gila monster (Heloderma suspectum) is a venomous lizard native to the Sonoran Desert. Despite significant activity in recovering a broad diversity of viruses associated with various reptiles (1) in the last decade, only viruses from the Adenoviridae family (2, 3) have been detected in Gila monsters to date. Here, we sampled the feces of a Gila monster to identify associated DNA viruses. The freshly deposited fecal sample (5 g) was homogenized in 20-ml SM buffer (0.1 M NaCl, 50 mM Tris HCl [pH 7.4]) and centrifuged at 10,000 × *g* for 10 min. The resulting supernatant was filtered sequentially through a 0.45-µm and 0.2-µm syringe filter. We used 200 µl of the filtrate to extract viral DNA using the High Pure viral nucleic acid kit (Roche Diagnostics, USA). Circular viral DNA was amplified using rolling circle amplification (RCA) with the Illustra TempliPhi 100 amplification kit (GE Healthcare, USA). The RCA products were used to prepare a 2 × 100-bp paired-end library and sequenced on a HiSeq 4000 instrument. Paired-end reads (24,473,832 reads, read length 101) were trimmed using Trimmomatic v0.36 (4) and then *de novo* assembled using ABySS 2.0 (5) with a k-mer of 64 resulting in 304,270 contigs (*N*_50_, 1,868 nucleotides). All *de novo* assembled contigs of >500 nucleotides (nt) were analyzed using a BLASTx search (6) against a local viral protein database compiled from GenBank. A contig of ∼2,000 nt was identified as having similarity to the replication-associated protein (Rep) encoded by viruses in the family Genomoviridae. Genomoviruses have small, circular, single-stranded DNA genomes that encode two main proteins, the Rep and a capsid protein (CP) (7). Genomoviruses (*n* = 196) have been detected from various samples, including plant leaves, blood, serum, cerebrospinal fluid, feces, and the whole body of various insects, wastewater, and sediments; however, only one genomovirus (Sclerotinia sclerotiorum hypovirulence-associated DNA virus 1) infecting the fungus Sclerotinia sclerotiorum has been identified (8).

Based on the genomovirus-like contig, a pair of abutting primers (Gila_283F, 5′-GTATTGTATCTAGCTAGCCTCATGCCAATATGTTG-3′, and Gila_283R, 5′-AAATTGGATCTATCACATTCACAATTACGCAGTTG-3′) were designed to recover the complete genome. The genome was amplified with PCR using the Gila_283F/R primer pair with KAPA HiFi HotStart DNA polymerase (Roche, USA), cloned, and Sanger sequenced.

The Gila monster-associated virus 1 (GmaV1; GenBank accession number MH378453) genome sequence is 2,191 nt (GC content, 50.5%), has a “TAATATTAT” nonanucleotide motif, and encodes three proteins, the CP in the virion sense and the Rep and RepA on the complementary sense. The GmaV1 genome shares 88%, 75%, and 66% genome pairwise identity, respectively, with a caribou feces-associated genomovirus sampled in Canada (GenBank accession number KJ938717) (9), a bovine blood-associated genomovirus from Germany (LK931484) (10), and a sewage-associated genomovirus from a sewage oxidation pond in New Zealand (KJ547634) (11). The CP and Rep amino acid sequences of GmaV1 share between 69 and 84% and 39 and 90% pairwise identity, respectively, with those of the genomoviruses from caribou feces (KJ938717) (9), bovine blood (LK931484) (10), and the sewage oxidation pond (KJ547634) (11). Based on the Genonoviridae classification (12), GmaV1 would belong to the genus Gemykrogvirus, which currently has three species. GmaV1 represents the third documented virus to be associated with the Gila monster; however, it is unknown whether this virus is associated with its gut flora or diet or whether it is pathogenic to the animal.

### Data availability.

The complete genome sequence of Gila monster-associated virus 1 has been deposited in GenBank with accession number MH378453.
